# Metabolic bone disease in extremely preterm infants: incidence, risk factors, and outcomes from a structured bone health program

**DOI:** 10.3389/fped.2025.1676540

**Published:** 2025-11-25

**Authors:** Saif Alsaif, Mohanned Alrahili, Talal Aljarbou, Lina Alsherbini, Mohammad Maghoula, Alanoud Alluwaymi, Mesaed Alsenani, Abdulrahman Altuwaym, Faisal Alamer, Abdulrahman Mandurah, Beverly Baylon, Ibrahim Ali, Kamal Ali

**Affiliations:** 1 Neonatal Intensive Care Department, King Abdulaziz Medical City-Riyadh, Ministry of National Guard Health Affairs, Riyadh, Saudi Arabia; 2 King Abdullah International Medical Research Center, Riyadh, Saudi Arabia; 3 College of Medicine, King Saud Bin Abdulaziz University for Health Sciences, Riyadh, Saudi Arabia

**Keywords:** metabolic, bone, disease, prematurity, outcome

## Abstract

**Background:**

Metabolic bone disease (MBD) of prematurity is a common disorder in extremely preterm and extremely low-birth-weight (ELBW) infants. However, regional data on this disorder from the Middle East are limited. We evaluated the incidence, risk factors, biochemical markers, and outcomes of MBD in infants born at <28 weeks of gestation and <1,000 g.

**Methods:**

Our retrospective cohort included 487 inborn preterm infants admitted to a tertiary NICU (Riyadh, Saudi Arabia; 2017–2024). MBD was defined as PTH >18 pmol/L at 4 weeks; ROC against radiographic osteopenia showed good discrimination (AUC 0.78). The clinical characteristics, nutrient intake, growth, and biochemical markers (ALP, phosphate, calcium, vitamin D, PTH) of the infants were analyzed. Logistic regression identified predictors and associations with adverse outcomes.

**Results:**

MBD was diagnosed in 202 out of 487 infants (41.5%). Compared with infants without MBD, those with MBD had lower GA and birth weight (both *p* < 0.001), more postnatal steroid exposure (44% vs. 27%, *p* < 0.001), longer diuretic therapy (12% vs. 3.5%, *p* < 0.001), and TPN beyond 28 days (50% vs. 31%, *p* < 0.001). PTH and ALP values were higher, while vitamin D, calcium, and magnesium concentrations were lower (all *p* < 0.01). Despite similar calcium/phosphate intakes, MBD was associated with postnatal growth failure (77% vs. 64%, *p* = 0.005), hospitalization of >60 days (88% vs. 70%, *p* < 0.001), and discharge on mineral supplements (36% vs. 16%, *p* < 0.001). Radiologic osteopenia occurred in 17.3% of MBD infants (7.1% overall), while fractures were uncommon (1.8% overall; 4.4% in MBD). On multivariable analysis, MBD independently predicted fractures (aOR: 8.3, 95% CI: 1.01–68.3), prolonged hospitalization (aOR: 1.9, 95% CI: 1.09–3.29), and growth failure (aOR: 1.63, 95% CI: 1.06–2.53).

**Discussion:**

Within this <28-week cohort, skeletal complications were less frequent than suggested by many reports, plausibly reflecting a structured bone health program (routine biochemical screening, optimized mineral delivery, and minimal handling). Findings support the incorporation of PTH alongside ALP for earlier detection and point to modifiable exposures (prolonged TPN, diuretics, and steroids) as targets for prevention. Prospective multicenter validation with standardized thresholds and imaging strategies is warranted.

**Conclusion:**

MBD is common in extremely preterm infants and is associated with growth failure and prolonged hospitalization. A 4-week PTH screen showed good discrimination for radiologic osteopenia (AUC 0.78), supporting its role within structured bone health care. As our findings are based on a biochemical definition, diagnostic thresholds require external validation.

## Introduction

Metabolic bone disease (MBD) is a common complication in premature infants, particularly in those weighing less than 1,500 g (VLBW) or under 1,000 g (ELBW) at birth. The condition is typically diagnosed during the early postnatal period, with incidence rates reported between 16% and 40% within the first 1.5–4 months of life (1). Most skeletal mineral deposition occurs in the third trimester, and preterm birth interrupts this process, leaving infants with insufficient calcium and phosphorus stores and dependent on early postnatal nutritional adequacy for bone health (2, 3).

Risk factors for MBD include delayed advancement of enteral feeding, prolonged dependence on total parenteral nutrition (TPN) (4), suboptimal mineral intake, exposure to loop diuretics, and limited mechanical loading due to immobility (1, 5–9). Among infants diagnosed with MBD, radiographic evidence of osteopenia, indicating reduced bone mineralization, is frequently reported, though the prevalence varies by study and region; one series noted osteopenia in up to 50% of affected infants (10). While less common, clinically evident fractures remain a significant complication, with a reported incidence of approximately 10% among preterm infants with MBD in a retrospective cohort (11).

Diagnosis of MBD commonly relies on biochemical screening, including serum alkaline phosphatase (ALP) (12, 13), serum phosphate (14), calcium, vitamin D, and parathyroid hormone (PTH) (15). Though elevated ALP and secondary hyperparathyroidism (reflected by elevated PTH) values are frequently used biochemical indicators, consensus on optimal thresholds for defining MBD is lacking (2). Radiographic confirmation via skeletal surveys can detect osteopenia or fractures, but imaging findings often lag behind biochemical abnormalities, making early detection challenging (2, 16).

Beyond its diagnostic challenges, MBD may also be associated with broader clinical consequences. Emerging evidence suggests that MBD may negatively influence neonatal outcomes beyond disturbances in bone mineralization. Studies have reported associations with prolonged hospitalization and impaired postnatal growth (1).

While high-quality data on MBD exist from North America and Europe, there remains a paucity of large cohort studies from the Middle East. In Saudi Arabia, where prematurity rates are significant and clinical practices in neonatal nutrition vary across institutions, few studies have comprehensively assessed the incidence, risk factors, and clinical outcomes of MBD in extremely preterm, extremely low-birth-weight infants.

Building on a structured bone health program established at a major tertiary neonatal intensive care unit (NICU) in Saudi Arabia, this study aims to investigate the incidence of MBD, defined as PTH >18 pmol/L at 4 weeks of age, in preterm infants born at <28 weeks of gestation and <1,000 g birth weight. We further evaluate the association of MBD with nutritional factors, including calcium and phosphate intake before and after screening, and clinical outcomes such as radiographically confirmed fractures, prolonged hospitalization (>60 days), and postnatal growth failure (PNGF). Our goal is to generate region-specific evidence to guide early diagnostic strategies and optimize nutritional management for preterm infants at risk of metabolic bone disease.

## Methods

This retrospective cohort study was conducted at King Abdulaziz Medical City (KAMC) in Riyadh, Saudi Arabia, a tertiary-level referral center with one of the largest NICUs in the region. The NICU has a level III designation with a capacity of 50 intensive care cots and receives approximately 900 admissions annually, including a high volume of extremely preterm and extremely low-birth-weight infants. The unit is supported by a multidisciplinary team comprising neonatologists, neonatal nurses, clinical pharmacists, and registered dietitians. A structured bone health program has been implemented at KAMC since 2017 and includes standardized screening, nutritional monitoring, and management protocols for infants at risk of MBD.

All inborn preterm infants born between January 2017 and December 2024 at less than 28 weeks of gestation and with birth weight below 1,000 g were eligible for inclusion in this study. Infants were excluded if they were outborn, had major congenital anomalies or chromosomal abnormalities, or died prior to the scheduled screening age for MBD. The bone health program at KAMC mandates routine biochemical screening at four weeks of chronological age, or earlier if clinically indicated, for all eligible infants. The biochemical screening panel includes serum parathyroid hormone (PTH), ALP, phosphate, calcium, and vitamin D. For this study, MBD was operationally defined as elevated PTH > 18 pmol/L measured at 4 weeks of chronological age. This value corresponds to the upper reference limit for preterm infants in our institutional laboratory, derived from internal population reference intervals in routinely screened NICU samples. As this internal range had not previously been validated against radiologic or densitometric outcomes, we conducted a receiver operating characteristic (ROC) analysis using radiologic osteopenia as the reference standard. This analysis demonstrated good discrimination of osteopenia by PTH (area under the curve = 0.784, 95% CI: 0.715–0.852; *p* < 0.001), with the optimal Youden-derived cutoff of 18 pmol/L, confirming that the institutional threshold provides balanced sensitivity and specificity. The chosen value is numerically equivalent to approximately 180 pg/mL, the level at which Moreira et al. (17) observed strong associations with biochemical and radiologic evidence of MBD in preterm infants. Elevated PTH results were interpreted alongside ALP and phosphate trends to improve specificity for disturbed bone metabolism.

In addition to biochemical screening, detailed nutritional data were systematically recorded for all infants as part of the bone health protocol. Parenteral nutrition (PN) is initiated shortly after birth and includes individualized calcium and phosphate supplementation. Expressed maternal breast milk is the preferred enteral feed and is initiated early and advanced gradually as tolerated. Human milk fortifier is introduced once enteral intake exceeds 150 mL/kg/day. Preterm formula is used when maternal milk is insufficient or unavailable. Vitamin D supplementation is routinely provided according to unit protocol. For the purposes of this study, daily intake of calcium and phosphate (mg/kg/day) from both parenteral and enteral sources was calculated before and after the screening time point. A ratio of post-screening to pre-screening mineral intake was also derived to assess the response in mineral supplementation following identification of abnormal biochemical markers.

Growth measurements were extracted from clinical records and converted to weight-for-age z-scores using the Fenton growth chart. Z-scores were documented at birth and again at 36 weeks of postmenstrual age. PNGF was defined as a decrease in weight-for-age z-score of ≥0.8 between birth and 36 weeks, reflecting clinically significant deviation from expected growth velocity. Radiographic fractures were identified through systematic review of radiology reports and were defined as confirmed long bone or rib fractures visible on standard radiographic imaging, whether identified incidentally or due to clinical suspicion. Prolonged hospitalization was defined as an NICU stay exceeding 60 days. Parenteral nutrition-associated cholestasis was defined as a conjugated bilirubin level of >34 μmol/L (2 mg/dL) persisting for more than 2 weeks in the absence of sepsis or known hepatic structural disease.

Data were collected from electronic medical records and included maternal and perinatal variables (hypertensive disorders, diabetes, antenatal steroid administration, mode of delivery, and multiple gestation), early neonatal variables (gestational age at birth, birth weight, postnatal steroid and diuretic exposure, and necrotizing enterocolitis), and detailed nutritional variables. No data were collected on bronchopulmonary dysplasia (BPD) in this study. The goal of data collection was to comprehensively characterize the clinical and nutritional context surrounding MBD diagnosis and to evaluate its association with adverse neonatal outcomes, particularly fractures, prolonged hospitalization, and postnatal growth failure.

All biochemical and clinical data were reviewed by the study team to ensure completeness and internal consistency before analysis. Data extraction was performed using a standardized form and reviewed independently by two investigators. The study was approved by the institutional review board of King Abdullah International Medical Research Center (IRB number: NRR25/163/5) with a waiver of informed consent due to its retrospective nature.

### Statistical analysis

All statistical analyses were conducted using IBM SPSS Statistics, Version 26.0 (IBM Corp., Armonk, NY, USA). The distribution of continuous variables was assessed using the Shapiro–Wilk test. Variables with a normal distribution were summarized as mean and standard deviation (SD), while non-normally distributed variables were reported as median and interquartile range (IQR). Categorical variables were described as counts and percentages.

To evaluate the discriminatory performance of PTH for radiologic osteopenia, a ROC analysis was performed using osteopenia (present/absent) as the state variable. The area under the curve (AUC) with 95% confidence intervals was calculated, and the Youden index was used to identify the optimal cutoff providing the best trade-off between sensitivity and specificity. Sensitivity and 1−specificity coordinates were inspected across the observed range of PTH values.

Comparisons between infants with and without MBD were performed using independent samples *t*-tests for normally distributed variables and Mann–Whitney *U*-tests for non-normally distributed variables. Categorical variables were compared using the Chi-square test or Fisher's exact test, as appropriate. Changes in weight-for-age z-scores from birth to 36 weeks of postmenstrual age were calculated using the Fenton growth chart, and PNGF was defined as a decline in z-score of ≥0.8 during this interval.

Multivariable logistic regression was used in two stages. (1) To identify independent predictors of MBD, candidate variables significant at *p* < 0.10 on univariate analysis and those deemed clinically plausible based on previous literature were considered. To maintain ≥10 events per variable, three covariates were retained in the final model: prolonged diuretic use (>2 weeks), prolonged parenteral nutrition (>28 days), and postnatal steroid exposure. Gestational age was evaluated for collinearity but not forced into this model. (2) A separate model was used to examine whether MBD independently predicted adverse outcomes (fractures, prolonged hospitalization > 60 days, and postnatal growth failure), adjusting for gestational age as a continuous covariate. Model fit was verified using the Hosmer–Lemeshow test. Results are reported as adjusted odds ratios (aOR) with 95% confidence intervals. Length of stay was further explored using Kaplan–Meier survival analysis to compare time to discharge between MBD and non-MBD groups. The log-rank test was used to evaluate the statistical significance of differences between survival curves. All statistical tests were two-tailed, and a *p*-value of <0.05 was considered statistically significant.

## Results

Table 1 summarizes the demographic and clinical characteristics of infants with and without MBD. The median gestational age at birth was lower in the MBD group compared to the non-MBD group [26 weeks (IQR 24–27) vs. 27 weeks (IQR 26–28); *p* < 0.001]. Similarly, the MBD group had a lower birth weight [800 g (IQR 625–1,000) vs. 920 g (IQR 770–1,100); *p* < 0.001]. There were no significant differences between the MBD and non-MBD groups in terms of male gender distribution [55% vs. 56%; *p* = 0.854], small-for-gestational-age status [3.5% vs. 4%; *p* = 0.438], cesarean delivery [58% vs. 61%; *p* = 0.538], multiple pregnancy [27% vs. 28%; *p* = 0.772], maternal diabetes [13% vs. 14%; *p* = 0.737], [800 g (IQR 625–1,000) vs. 920 g (IQR 770–1,100); *p* = 0.737], antenatal steroid exposure [81% vs. 83%; *p* = 0.617], or maternal hypertension [9% vs. 11%; *p* = 0.230]. Postnatal steroid use was more frequent in the MBD group [44% vs. 27%; *p* < 0.001]. Use of diuretics for more than two weeks was higher in the MBD group [12% vs. 3.5%; *p* < 0.001], as was prolonged parenteral nutrition exceeding 28 days [50% vs. 31%; *p* < 0.001] (Table 1).

**Table 1:** Perinatal and neonatal characteristics of infants with and without MBD.

Variable-median (IQR), %	MBD group (*n* = 202)	Non-MBD group (*n* = 285)	*p*-Value
GA at birth (weeks)	26 [24,27]	27 [26,28]	<0.001
Male gender	55	56	0.854
Birth weight (g)	800 [625,1,000]	920 [770,1,100]	<0.001
SGA status	3.5	4	0.438
Cesarean delivery	58	61	0.538
Multiple pregnancy	27	28	0.772
Maternal hypertension	9	11	0.230
Maternal diabetes	13	14	0.737
Maternal antenatal steroids	81	83	0.617
Postnatal steroids	44	27	<0.001
Diuretics therapy > 2weeks	12	3.5	<0.001
TPN > 28 days	50	31	<0.001

Table 2 presents a comparison of biochemical parameters between infants with and without MBD. Infants in the MBD group had significantly higher ALP levels at screening [410 IU/L (SD 146) vs. 365 IU/L (SD 150); *p* = 0.001] and higher peak ALP levels [600 IU/L (SD 205) vs. 524 IU/L (SD 194); *p* < 0.001]. Both screening and peak PTH concentrations were markedly elevated in the MBD group [29 pmol/L (SD 10.4) vs. 11 pmol/L (SD 4.2); *p* < 0.001 and 31.5 pmol/L (SD 15.5) vs. 14.2 pmol/L (SD 8.1); *p* < 0.001, respectively]. Serum phosphate levels were not significantly different between groups [1.86 mmol/L (SD 0.33) vs. 1.91 mmol/L (SD 0.32); *p* = 0.166]. However, infants with MBD had significantly lower serum calcium [2.5 mmol/L (SD 0.14) vs. 2.6 mmol/L (SD 0.14); *p* < 0.001] and magnesium levels [0.78 mmol/L (SD 0.09) vs. 0.81 mmol/L (SD 0.08); *p* < 0.001], as well as lower vitamin D concentrations [53 nmol/L (SD 20) vs. 60 nmol/L (SD 20); *p* = 0.001] (Table 2).

**Table 2:** Comparison of biochemical markers between infants with and without MBD.

Variable-mean (SD)	MBD group (*n* = 202)	Non-MBD group (*n* = 285)	*p*-Value
ALP at screening (IU/L)	410 (146)	365 (150)	0.001
Peak ALP (IU/L)	600 (205)	524 (194)	<0.001
PTH at screening (pmol/L)	29 (10.4)	11 (4.2)	<0.001
Peak PTH (pmol/L)	31.5 (15.5)	14.2 (8.1)	<0.001
Phosphate (mmol/L)	1.86 (0.33)	1.91 (0.32)	0.166
Calcium (mmol/L)	2.5 (0.14)	2.6 (0.14)	<0.001
Magnesium (mmol/L)	0.78 (0.09)	0.81 (0.08)	<0.001
Vitamin D (nmol/L)	53 (20)	60 (20)	0.001

### Diagnostic validation of PTH for radiologic osteopenia

ROC analysis demonstrated good discrimination of radiologic osteopenia by PTH, with an area under the curve (AUC) of 0.784 (95% CI: 0.715–0.852; *p* < 0.001). The optimal Youden index corresponded to a cutoff of approximately 18 pmol/L, confirming that this threshold provides a balanced trade-off between sensitivity and specificity for detecting infants with radiologic osteopenia. These findings validate the use of the institutional upper reference limit as a clinically meaningful threshold for secondary hyperparathyroidism and biochemical MBD screening. The ROC curve is provided in Supplementary Figure S1.

Table 3 presents the growth and nutritional characteristics of infants with and without MBD. Infants in the MBD group had significantly lower birth weights [800 g (IQR 625–1,000) vs. 920 g (IQR 770–1,100); *p* < 0.001], although there was no significant difference in birth z-score [0.25 (IQR −0.47 to 0.79) vs. 0.34 (IQR −0.39 to 0.83); *p* = 0.509]. Weight at 36 weeks of postmenstrual age was comparable between groups [2,065 g (IQR 1,862–2,267) vs. 2,025 g (IQR 1,842–2,238); *p* = 0.599], as were z-scores at 36 weeks [–1.32 (IQR −1.8 to −0.8) vs. −1.34 (IQR −1.9 to −0.7); *p* = 0.766]. PNGF was significantly higher among infants with MBD [77% vs. 64%; *p* = 0.005]. There was no significant difference in the time to start enteral feeding [3 days (IQR 2–5) vs. 3 days (IQR 2–4); *p* = 0.874] or the time to reach full feeds [20 days (IQR 14–30) vs. 18 days (IQR 13–30); *p* = 0.091] between the two groups.

**Table 3:** Growth metrics, nutritional intake, and parenteral nutrition characteristics in infants with and without MBD.

Variable-median (IQR), %	MBD group (*n* = 202)	Non-MBD group (*n* = 285)	*p*-Value
Birth weight (g)	800 [625,1,000]	920 [770,1,100]	<0.001
Birth z-score	0.25 [−0.47,0.79]	0.34 [−0.39,083]	0.509
Weight at 36 weeks (g)	2,065 [1,862,2,267]	2,025 [1,842,2,238]	0.599
36w z-score	−1.32 [−1.8, −0.8]	−1.34 [−1.9, −0.7]	0.766
PNGF (Δz ≥ 0.8)	77	64	0.005
Time to start enteral feeding	3 [2,5]	3 [2,4]	0.874
Time to full feeds (days)	20 [14,30]	18 [13,30]	0.091
TPN duration (days)	28 [16,36]	19 [12,24]	0.001
TPN dependency > 28 days	50	31	<0.001
TPN cholestasis	19	12	0.031
Calcium intake before MBD screening (mg/kg/day)	127 [82,164]	122 [80,162]	0.603
Phosphate intake before MBD screening (mg/kg/day)	82 [62,103]	78 [58,97]	0.219
Calcium: phosphate ration before MBD screening	1.58 [1.3,1.7]	1.60 [1.3,1.7]	0.452
Calcium intake after MBD screening (mg/kg/day)	149 [106,183]	149 [100,180]	0.992
Phosphate intake after MBD screening (mg/kg/day)	90 [73,106]	92 [68,107]	0.875
Calcium: phosphate ration after MBD screening	1.63 [1.4,1.7]	1.65 [1.4,1.8]	0.869

Duration of TPN was significantly longer in the MBD group [28 days (IQR 16–36) vs. 19 days (IQR 12–24); *p* = 0.001], and a greater proportion of these infants were dependent on TPN for more than 28 days [50% vs. 31%; *p* < 0.001]. TPN-associated cholestasis was also more frequent in the MBD group [19% vs. 12%; *p* = 0.031].

No statistically significant differences were observed in pre- or post-screening calcium intake [pre: 127 mg/kg/day (IQR 82–164) vs. 122 mg/kg/day (IQR 80–162); *p* = 0.603, post: 149 mg/kg/day (IQR 106–183) vs. 149 mg/kg/day (IQR 100–180); *p* = 0.992], phosphate intake [pre: 82 mg/kg/day (IQR 62–103) vs. 78 mg/kg/day (IQR 58–97); *p* = 0.219, post: 90 mg/kg/day (IQR 73–106) vs. 92 mg/kg/day (IQR 68–107); *p* = 0.875], or calcium-to-phosphate ratios before [1.58 (IQR 1.3–1.7) vs. 1.60 (IQR 1.3–1.7); *p* = 0.452] and after [1.63 (IQR 1.4–1.7) vs. 1.65 (IQR 1.4–1.8); *p* = 0.869] screening (Table 3).

Table 4 compares the frequency of adverse outcomes between infants with and without MBD. Fractures occurred more frequently among infants with MBD [4% vs. 0.4%; *p* = 0.004], as did radiologically confirmed osteopenia [16% vs. 1%; *p* < 0.001]. There was no significant difference in the incidence of severe intraventricular hemorrhage (IVH grades 3–4) [15% vs. 13%; *p* = 0.418] or surgical necrotizing enterocolitis (NEC) [6% vs. 5%; *p* = 0.728].

**Table 4:** Comparison of adverse clinical outcomes between infants with and without MBD.

Outcome-percentage	MBD group (*n* = 202)	Non-MBD group (*n* = 285)	*p*-Value
Fractures	4	0.4	0.004
Osteopenia	16	1	<0.001
Severe IVH (Grade 3–4)	15	13	0.418
Surgical NEC	6	5	0.728
PNGF	77	64	0.005
Mortality	2.5	2.5	0.991
LOS > 60 days	88	70	<0.001
Discharged on calcium and phosphate supplements	36	16	<0.001

The incidence of PNGF was significantly higher in the MBD group [77% vs. 64%; *p* = 0.005], and a greater proportion of infants with MBD experienced prolonged hospitalization, defined as length of stay greater than 60 days [88% vs. 70%; *p* < 0.001]. Discharge on calcium and phosphate supplementation was also more common among MBD infants [36% vs. 16%; *p* < 0.001]. There was no difference in overall mortality between the groups [2.5% vs. 2.5%; *p* = 0.991] (Table 4).

Multivariable logistic regression analysis was conducted to identify independent predictors of MBD among preterm infants (Table 5). Variables with *p* < 0.10 on univariate analysis and those considered biologically plausible were entered into the model. Three variables remained independently associated with MBD: prolonged diuretic exposure (>2 weeks; aOR: 2.53, 95% CI: 1.15–5.60, *p* = 0.022), total parenteral nutrition > 28 days (aOR: 1.60, 95% CI: 1.06–2.42, *p* = 0.027), and postnatal steroid use (aOR: 1.58, 95% CI: 1.04–2.41, *p* = 0.033). The model demonstrated acceptable calibration (Hosmer–Lemeshow *p* = 0.71) and moderate explanatory power (Nagelkerke *R*^2^ = 0.14). These findings suggest that prolonged exposure to catabolic medications and delayed nutritional autonomy are key risk factors contributing to impaired bone mineralization in this population (Table 5).

**Table 5:** Multivariable logistic regression: predictors of metabolic bone disease.

Predictor	aOR	95% CI	*p*-Value
Diuretic use > 2 weeks	2.53	1.15–5.60	0.022
TPN > 28 days	1.60	1.06–2.42	0.027
Postnatal steroids	1.58	1.04–2.41	0.033

Model adjusted for variables significant on univariate analysis (*p* < 0.10) and selected for biological plausibility. Hosmer–Lemeshow *p* = 0.71; Nagelkerke *R*^2^ = 0.14.

Multivariable logistic regression was performed to examine the association between MBD and major adverse outcomes, with adjustment for gestational age (Table 6). After controlling for gestational age, MBD remained independently associated with a higher likelihood of radiographically confirmed fractures (aOR: 8.30, 95% CI: 1.01–68.26, *p* = 0.049), prolonged hospitalization exceeding 60 days (aOR: 1.90, 95% CI: 1.09–3.29, *p* = 0.023), and postnatal growth failure (aOR: 1.63, 95% CI: 1.06–2.53, *p* = 0.028). Gestational age itself was inversely related to these adverse outcomes, with each additional week of maturity reducing the odds (aOR: 1.25, 95% CI: 1.10–1.42, *p* = 0.001). The model demonstrated good calibration (Hosmer–Lemeshow *p* = 0.64). These findings underscore that, even after accounting for prematurity, MBD is an independent risk factor for prolonged hospitalization, poor postnatal growth, and fractures in extremely preterm infants (Table 6).

**Table 6:** Multivariable logistic regression: association between MBD and adverse outcomes (adjusted for gestational age).

Outcome	aOR	95% CI	*p*-Value
Fractures	8.30	1.01–68.26	0.049
LOS > 60 days	1.90	1.09–3.29	0.023
Postnatal growth failure	1.63	1.06–2.53	0.028
Gestational age (per week)	1.25	1.10–1.42	0.001

Model includes MBD status and gestational age as covariates; Hosmer–Lemeshow *p* = 0.64.

**Figure 1:**
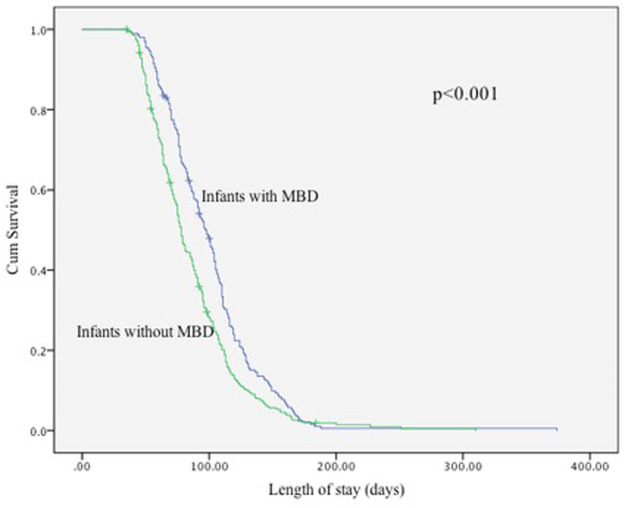
Kaplan–Meier curve for length of stay of infants with and without MBD.

Kaplan–Meier analysis demonstrated significantly longer hospital stays among infants with MBD compared with those without MBD (Figure 1, Table 7). The median length of stay was 97 days (95% CI: 89.6–104.4) for infants with MBD and 78 days (95% CI: 74.4–81.6) for those without MBD. Global tests confirmed a significant difference between the survival distributions (log-rank *χ*^2^ = 14.0, *p* < 0.001; Breslow *χ*^2^ = 23.8, *p* < 0.001; Tarone–Ware *χ*^2^ = 21.1, *p* < 0.001).

**Table 7:** Number of infants at risk at selected time intervals and summary survival statistics from the Kaplan–Meier analysis of hospital stay in infants with and without MBD.

Length of stay (days)	0	50	100	150	200
Infants with MBD (*n* = 200)	200	193	93	50	20
Infants without MBD (*n* = 275)	275	225	175	125	3

Group	Median length of stay (days)	95% CI
Summary survival statistics		
Infants with MBD (*n* = 200)	97	(89.6–104.4)
Infants without MBD (*n* = 275)	78	(74.4–81.6)
Global tests	Log-rank *χ*² = 14.0, *p* < 0.001; Breslow *χ*² = 23.8, *p* < 0.001; Tarone–Ware *χ*² = 21.1, *p* < 0.001	

Numbers at risk reflect infants still hospitalized at each time point; decreases reflect discharges or censoring.

## Discussion

In this large retrospective cohort of extremely preterm infants enrolled in a structured bone health program, we evaluated the incidence of MBD and its association with key neonatal outcomes. Using a biochemically defined threshold of parathyroid hormone (PTH) > 18 pmol/L, validated in this cohort by receiver operating characteristic analysis (AUC = 0.78), we identified significant differences in biochemical markers between affected and unaffected infants. MBD was independently associated with a higher risk of fractures, postnatal growth failure, and prolonged hospitalization. In addition, prolonged diuretic exposure, extended parenteral nutrition, and postnatal steroid therapy were significant predictors of MBD development.

The development of MBD of prematurity is closely linked to the degree of prematurity at birth. Infants born at earlier gestational ages and with lower birth weights have a higher risk of developing MBD. Reported incidence rates reach up to 30% among infants born before 28 weeks of gestation, and approximately 10% of preterm infants experience fractures by 36–40 weeks of corrected gestational age (18, 19). Consistent with this finding, our study also identified lower gestational age as a significant risk factor for the development of MBD. The primary mechanism is thought to be the inadequate accumulation of mineral stores due to premature delivery. The third trimester, particularly between 25 and 40 weeks of gestation, is a critical period during which the fetus accrues approximately 80% of its total calcium and phosphorus stores (4, 20, 21). Preterm birth during this window may significantly disrupt this process, resulting in reduced mineral reserves at birth.

Infants diagnosed with MBD in our cohort demonstrated significantly higher levels of both screening and peak ALP and PTH levels compared to infants without MBD, highlighting their utility as early biochemical markers of disturbed bone metabolism. These elevations align with secondary hyperparathyroidism driven by inadequate mineral supply, particularly calcium (22). Although serum calcium and magnesium levels were statistically lower in the MBD group, the absolute values in both groups remained within the normal reference range. This suggests that even modest shifts within the normal range may be physiologically significant in extremely preterm infants, triggering compensatory hormonal responses such as elevated PTH. Notably, serum phosphate levels were comparable between groups, indicating that phosphate deficiency alone may not have been a primary driver of MBD in this cohort. These findings support the use of ALP and PTH as sensitive markers of early mineral imbalance, even in the absence of overt hypocalcemia, hypomagnesemia, or hypophosphatemia.

Several studies have shown that vitamin D status in preterm infants is strongly influenced by gestational age, with lower 25-hydroxyvitamin D [25(OH)D] levels observed in those born at earlier gestations. One cohort found that infants with lower vitamin D concentrations at birth had increased respiratory support needs, including greater oxygen requirements and longer durations of mechanical ventilation (23). In addition, a large randomized trial demonstrated that vitamin D supplementation at doses of 800–1,000 IU/day effectively reduced deficiency rates at 36 weeks of postmenstrual age in preterm infants ≤32 weeks (24). These findings are consistent with our own study, where vitamin D concentrations measured at the screening age of 4 weeks were significantly lower in infants diagnosed with MBD. In our cohort, infants who met the MBD criteria had lower 25(OH)D levels despite receiving standardized supplementation. This suggests that routine dosing may be insufficient for extremely preterm infants and supports the potential value of incorporating vitamin D monitoring into screening protocols to guide individualized supplementation and reduce the risk of MBD.

In our cohort, prolonged diuretic exposure (>2 weeks) was associated with more than double the odds of MBD (aOR: 2.53, 95% CI: 1.15–5.60, *p* = 0.022), consistent with the known effects of loop diuretics on urinary calcium loss and bone demineralization. Prolonged TPN (>28 days) was also associated with higher odds of MBD (aOR: 1.60, 95% CI: 1.06–2.42, *p* = 0.027), plausibly reflecting limited enteral tolerance and suboptimal mineral delivery during a critical growth window. Postnatal corticosteroid therapy likewise increased MBD risk (aOR: 1.58, 95% CI: 1.04–2.41, *p* = 0.033). These associations align with prior reports: Cumulative furosemide ≥8 mg/kg was linked to higher MBD incidence (25), and in infants with BPD, diuretic use of >2 weeks carried an adjusted OR ≈ 5.5 (95% CI: 1.25–23.8; *p* < 0.05) (26); broader reviews also identify diuretics and steroids as modifiable contributors of MBD in extremely low-birth-weight infants (2, 27). Mechanistically, loop diuretics promote urinary calcium loss and stimulate PTH, while corticosteroids impair osteoblast function and reduce intestinal calcium absorption (20). Taken together, our multivariable model and the external literature support prioritizing early identification of high-risk infants and implementing close biochemical monitoring with individualized mineral support for those on prolonged diuretics, extended parenteral nutrition, or systemic corticosteroids.

Beyond biochemical and clinical parameters, our study also examined growth metrics and nutritional exposures in relation to MBD. We observed that while weight at 36 weeks and corresponding z-scores were similar between the groups, the incidence of postnatal growth failure was significantly higher among infants with MBD. This suggests that growth trajectory was negatively affected despite similar weight outcomes later in hospitalization, likely due to early nutritional deficits and increased catabolic stress. Infants with MBD in our cohort had longer durations of total parenteral nutrition and were more frequently dependent on TPN beyond 28 days. TPN-associated cholestasis was also more prevalent in this group, reflecting delayed advancement of enteral feeds and prolonged exposure to parenteral nutrition.

In our cohort, there were no significant differences in calcium or phosphate intake between infants with and without MBD, both before and after the time of biochemical screening. Furthermore, the calcium-to-phosphate intake ratios in both groups remained within the recommended range of 1.5:1 to 1.7:1 (mg:mg) before and after MBD screening, a balance considered optimal for promoting phosphate retention and calcium absorption (28). Despite these appropriate ratios, MBD still occurred in some infants, suggesting contributions from factors beyond intake, such as variability in absorption, intercurrent illness, or delays in supplementation. These findings support meeting nutritional targets while also monitoring biochemical markers closely to identify infants at risk.

The comparison of adverse clinical outcomes in our cohort demonstrates the clinical burden associated with MBD in preterm infants. The higher frequency of osteopenia and fractures in the MBD group highlights the potential skeletal consequences of inadequate postnatal mineralization. These findings align with the pathophysiology of MBD, in which insufficient calcium and phosphate accretion leads to impaired bone strength and reduced structural integrity. Although no significant differences were observed in other major complications such as severe intraventricular hemorrhage or surgical necrotizing enterocolitis, the presence of MBD was associated with a significantly greater likelihood of postnatal growth failure and prolonged hospitalization, both of which are important indicators of neonatal morbidity and complexity of care. The increased length of stay observed among infants with MBD may reflect the cumulative impact of delayed enteral feeding, prolonged parenteral nutrition, and persistent metabolic instability. Importantly, while mortality rates were similar between the two groups, the higher rates of morbidity among infants with MBD suggest that the condition may contribute to prolonged illness and increased healthcare resource use, even in the absence of increased mortality risk.

In our cohort, radiologically confirmed osteopenia occurred in 7.1% of all infants and in 17.3% of those meeting the biochemical definition MBD. Fractures were uncommon overall (1.8%) but more frequent among infants with radiological osteopenia (25.7%) and among those with biochemical MBD (4.4%). By comparison, prior reports describe osteopenia in 30%–50% of extremely low-birth-weight infants and up to 40% of very low-birth-weight infants (<1,500 g) (5, 29). Cross-sectional data in infants <32 weeks have shown radiographic MBD in 9.4% and biochemical MBD in 12.5% based on alkaline phosphatase and phosphate thresholds (30), and larger retrospective series report radiologic MBD in up to 50% of extremely low-birth-weight infants, with 30%–40% among those <1,000 g (10).

Our lower fracture rate relative to some studies may reflect, in part, structured surveillance in our unit (scheduled biochemical screening with PTH and ALP, standardized nutritional audits, and routine radiology rather than symptom-triggered imaging) and local practices such as minimal handling. Other explanations are also plausible, including differences in case mix, diagnostic thresholds, timing of radiographs, ascertainment methods, and secular improvements in neonatal care. As comparator group was included, these findings are descriptive and do not permit causal attribution of fracture outcomes to the bone health program.

A major strength of this study is the large cohort size, which is among the largest reported datasets examining MBD in extremely preterm infants. The inclusion of 487 infants over an 8-year period enhances the robustness and generalizability of the findings. Importantly, the study focused exclusively on infants born at <28 weeks of gestation and <1,000 grams birth weight, a population at highest risk for MBD, yet often underrepresented in large-scale studies. Another significant strength is the integration of all infants within a structured bone health program. This allowed for uniform biochemical screening at a defined time point, systematic nutritional monitoring, and routine radiological surveillance, reducing detection bias and improving diagnostic consistency. The use of PTH as a central diagnostic marker adds further clinical relevance, given the growing recognition of PTH as a sensitive early indicator of mineral homeostasis disturbance. Detailed data on mineral intake, growth parameters, and neonatal outcomes enhance the comprehensiveness and clinical applicability of the findings. However, this study is not without limitations. Its retrospective design limits the ability to establish causality, and residual confounding from unmeasured clinical variables cannot be excluded. The diagnosis of MBD was based on biochemical criteria, without gold-standard imaging modalities such as dual-energy x-ray absorptiometry, which may have resulted in misclassification in borderline cases. The PTH >18 pmol/L threshold, originally based on our institutional reference limit and numerically close to ≈180 pg/mL reported by Moreira et al., was internally validated against radiologic osteopenia (AUC 0.784, 95% CI: 0.715–0.852). While this supports its use as a marker of secondary hyperparathyroidism, external multicenter validation is still needed to establish universal diagnostic criteria. The study included only inborn infants, which may introduce selection bias. Although 21 outborn infants admitted during the study period would otherwise have met inclusion criteria, most were transferred to our center after the 4-week screening age and were therefore excluded. This restriction ensured consistent timing of laboratory and radiologic evaluations but may have led to a modest underestimation of the overall incidence of MBD and limits generalizability to transferred populations, who often have differing nutritional exposures and illness severity before referral. Alongside these design constraints, we lacked data on BPD, a common comorbidity often treated with diuretics and corticosteroids; consequently, we could not adjust for BPD, and residual confounding may inflate or attenuate the observed drug–MBD associations.

Given the limitations of the study, future studies should use a multicenter prospective design, capture BPD and detailed diuretic/steroid exposures, standardize nutritional intakes, and pair biochemical screening with a uniform imaging protocol. Future studies should also validate the PTH cutoff against a composite MBD definition, report imaging dose, explore quantitative ultrasound, and extend follow-up to 12–24 months to assess post-discharge skeletal outcomes.

## Conclusion

In this large single-center cohort of extremely preterm infants enrolled in a structured bone health program, metabolic bone disease was identified in over 40% of infants and was independently associated with postnatal growth failure, prolonged hospitalization, and fractures. Despite the high-risk population, the observed rates of osteopenia and fractures were lower than in prior reports, likely reflecting systematic biochemical screening, optimized mineral intake, and proactive radiologic surveillance. A 4-week PTH screen, interpreted with ALP, showed good discrimination for radiologic osteopenia (AUC 0.78). These findings support the implementation of standardized bone health protocols with early screening in NICUs caring for extremely preterm infants, while acknowledging that biochemical thresholds require external validation and standardization.

## Data Availability

The raw data supporting the conclusions of this article will be made available by the authors, without undue reservation.
