# Supplementary figures and images for: Metabolic bone disease in extremely preterm infants: incidence, risk factors, and outcomes from a structured bone health program
Source: Front Pediatr. 2025 Nov 25;13:1676540. doi: 10.3389/fped.2025.1676540 (PMC12685821; doi:10.3389/fped.2025.1676540)

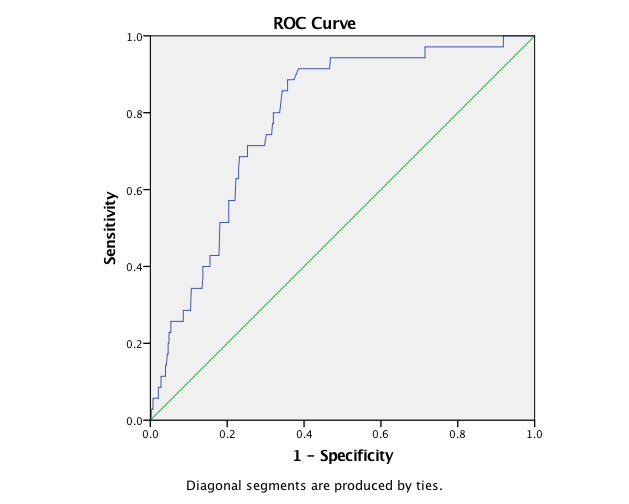

Supplement: Supplementary file 1 [file Image1.png]
